# The Relationship between Tricuspid Annular Longitudinal and Sphincter-Like Features of Its Function in Healthy Adults: Insights from the MAGYAR-Healthy Study

**DOI:** 10.3390/life13102079

**Published:** 2023-10-18

**Authors:** Attila Nemes, Gergely Rácz, Árpád Kormányos, Zoltán Ruzsa, Alexandru Achim, Csaba Lengyel

**Affiliations:** Department of Medicine, Albert Szent-Györgyi Medical School, University of Szeged, H-6720 Szeged, Hungarykormanyos.arpad@med.u-szeged.hu (Á.K.); zruzsa25@gmail.com (Z.R.); dr.alex.achim@gmail.com (A.A.); lecs@in1st.szote.u-szeged.hu (C.L.)

**Keywords:** echocardiography, healthy, speckle tracking, three-dimensional, tricuspid annulus

## Abstract

Introduction. The tricuspid valve is an atrioventricular valve located on the right side of the heart, which consists of the fibrous tricuspid annulus (TA), three valvular leaflets and a supporting apparatus, the papillary muscles and the tendinous chords. The TA is an oval-shaped three-dimensional (3D) fibrous structure with a complex spatial movement during the cardiac cycle. Three-dimensional echocardiography (3DE) could help during “en-face” assessment of TA dimensions and related functional properties featuring its “sphincter-like” function. TA plane systolic excursion (TAPSE) is a displacement of the lateral edge of the TA toward the apex in systole measured in apical long-axis using M-mode echocardiography (MME). The aim of this study was to determine potential relationships between TA size and its “sphincter-like” and “longitudinal” functions in healthy adults with no functional tricuspid regurgitation. Methods. The present study consisted of 119 healthy patients (age: 34.6 ± 11.5 years, 70 men) who underwent routine echocardiography with M-mode-derived TAPSE measurement and 3DE. Two subgroups of healthy subjects were compared with each other. A total of 29 subjects with TAPSE between 17 and 21 mm were compared with 90 cases with TAPSE ≥ 22 mm. Results. Subjects with TAPSE of 17–21 mm had tendentiously dilated TA dimensions compared with subjects with TAPSE ≥ 22 mm. Significant differences could be detected in the end-systolic TA area (5.85 ± 1.90 cm^2^ vs. 3.70 ± 1.22 cm^2^, *p* < 0.05), leading to impaired TAFAC (24.8 ± 9.0% vs. 35.1 ± 9.1%, *p* < 0.05) in subjects with lower TAPSE (17–21 mm) compared with subjects with TAPSE ≥ 22 mm. TAPSE did not show correlations with any TA size or “sphincter-like” functional parameters as determined using 3DE. Conclusions. Three-dimensional echocardiography is capable of measuring TA dimensions and functional “sphincter-like” properties, which are associated with MME-derived TAPSE, suggesting a sensitive and harmonic TA function in healthy adults without functional tricuspid regurgitation.

## 1. Introduction

Evaluation of the right side of the heart has come to the forefront of scientific thinking in recent years. The reason for this is twofold: on the one hand, new therapeutic procedures have become clinically usable that can be used for many disorders affecting the right side of the heart (e.g., congenital heart disease, pulmonary hypertension, etc.). On the other hand, non-invasive examination options that are easy to learn and use and enable a detailed and extensive analysis have become widespread in routine clinics, e.g., three-dimensional echocardiography (3DE) [1,2,3,4,5,6,7].

The tricuspid valve (TV) is an atrioventricular valve located on the right side of the heart, which consists of the fibrotic tricuspid annulus (TA), three valvular leaflets and a supporting apparatus, the papillary muscles and the tendinous chords [1]. The TV is responsible for the one-way flow of blood from the right atrium (RA) to the right ventricle (RV) without substantial regurgitation. While examining the TV was difficult in the past, new imaging options such as 3DE allow it to be examined in detail [4,5,6,7,8,9,10,11]. Therefore, every year, many studies are conducted focusing on the non-invasive evaluation of the TV.

Functional tricuspid regurgitation (FTR) is commonly a result of different cardiac diseases affecting the left heart with induced RV dilation and functional abnormalities, but it can be a result of a dilated RA and TA [1,2,3]. The TA is an oval-shaped three-dimensional (3D) fibrous structure with a complex spatial movement according to the heart cycle [1]. 3DE could help us understand the clinical implications of FTR, enabling “en-face” assessment of TA dimensions and related functional properties featuring its “sphincter-like” function [4,5,6,7,8,9,10,11]. However, the complexity of the TA function includes its longitudinal movement, which is well represented by its systolic excursion (TA plane systolic excursion, TAPSE), which can easily be determined via its M-mode-derived measurement [12,13,14,15,16]. A harmonic 3D movement of the TA could be theorized in healthy subjects before FTR develops. Therefore, due to limited quantitative information, the current study aimed to determine possible relationships between TA size and its “sphincter-like” and “longitudinal” functions in healthy adults without FTR.

## 2. Materials and Methods

Subjects: The present retrospective cohort study consisted of 154 healthy subjects who participated as a volunteer between 2011 and 2015, and the investigation was performed at our department. From this pool of healthy subjects, 35 individuals did not participate in this study due to image quality problems for 3DE measurements and/or M-mode assessment of TA plane systolic excursion (TAPSE). The remaining population was 119 subjects with a mean age of 30.1 ± 10.3 years (70 males). Their clinical parameters were within normal ranges, including weight (71.3 ± 12.3 kg), height (171.0 ± 10.2 cm), body surface area (1.84 ± 0.20 m^2^), body mass index (22.9 ± 3.2 kg/m^2^), systolic blood pressure (122.1 ± 4.6 mm Hg) and diastolic blood pressure (75.9 ± 4.1 mm Hg). An individual was considered healthy if they did not have acute or chronic illnesses in their medical history or showed ECG abnormality. Complete two-dimensional (2D) Doppler echocardiography was performed on all subjects and showed normal results. None of them were smokers or had a history of regular drug use. According to guidelines, TAPSE is considered to be normal if ≥17 mm, while the mean value of the TAPSE of healthy subjects was found approximately 21.5 mm in a recent study [12,13]. The group of healthy subjects was divided into 2 subgroups: subjects with TAPSE between 17 and 21 mm were compared with subjects with TAPSE ≥ 22 mm. All healthy volunteers underwent a complete M-mode, 2D Doppler echocardiographic and 3DE examination. This was a substudy of the Motion Analysis of the heart and Great vessels bY three-dimensionAl speckle-tRacking echocardiography in Healthy subjects (MAGYAR-Healthy) Study (“Magyar” means “Hungarian” in the Hungarian language). The study met the requirements of the Declaration of Helsinki (as revised in 2013 and the updated versions) and was approved by the Institutional and Regional Human Biomedical Research Committee of University of Szeged, Hungary, under the registration number 71/2011 and updated versions. All subjects provided informed consent.

**Figure 1:**
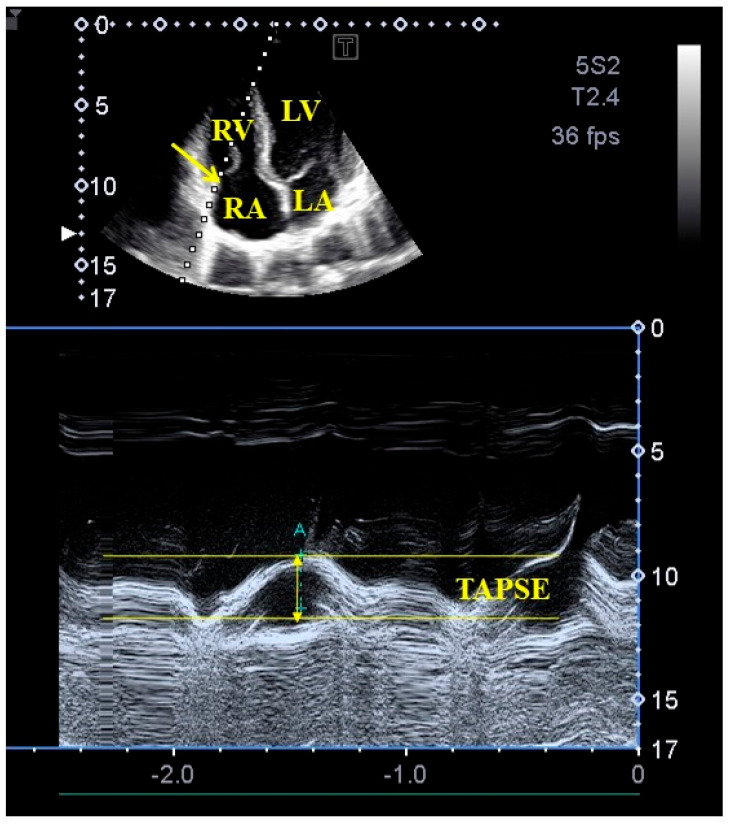
M-mode echocardiography-derived assessment of tricuspid annular plane systolic excursion (TAPSE) from apical four-chamber view. Abbreviations: LA = left atrium, LV = left ventricle, RA = right atrium, RV = right ventricle, TAPSE = tricuspid annular plane systolic excursion.

M-mode and two-dimensional Doppler echocardiography: Two-dimensional Doppler echocardiographic examinations were performed in accordance with available professional guidelines and accepted practices. During the examinations, a Toshiba Artida^TM^ echocardiography device was used, which could be connected to a PST-30BT (1–5 MHz) phased-array transducer. The person to be examined was asked to lie on their left side, and then, placing the transducer on the chest, measurements were taken from the typical sections from both the parasternal and apical directions. After performing left atrial and left ventricular measurements, the extent of any valvular regurgitation was determined using the continuous-wave Doppler method and visual estimation. Doppler echocardiography was used to exclude significant valvular stenosis as well. LV-EF was determined using Simpson’s method. Representing systolic longitudinal motion of the TA, TAPSE was measured in apical long-axis as a displacement of the lateral edge of the TA toward the apex in systole (Figure 1) [12,17,18].

**Figure 2:**
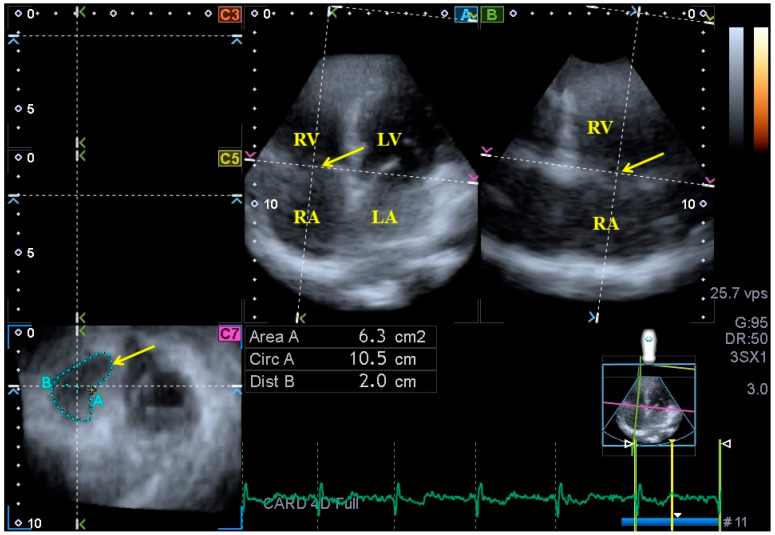
Assessment of the tricuspid annulus extracted from a three-dimensional full-volume dataset is presented: apical four-chamber view (**A**); apical two-chamber view (**B**); and a cross-sectional view at the level of the tricuspid annulus optimized in apical four- and two-chamber views (C7). The yellow arrow represents the tricuspid annular plane. Abbreviations: LA = left atrium, LV = left ventricle, RA = right atrium, RV = right ventricle, Area = TA area, Circ = TA perimeter, Dist = TA diameter.

Three-dimensional echocardiography. The aforementioned Toshiba Artida^TM^ cardiac ultrasound equipment attached to a PST-25SX matrix array transducer was used for 3DE examinations [5,6,7]. In accordance with our routines, pyramid-shaped 3D echocardiographic datasets were acquired from the apical window. Data were collected during six constant RR intervals seen on the ECG and during one breath hold, and an offline analysis was performed with the vendor-provided 3D Wall Motion-Tracking software (Ultra Extend, Toshiba Medical Systems, Tokyo, Japan, version 2.7) at a later date. Using the abovementioned 3D datasets, the software automatically selected apical two- (AP2CH) and four-chamber (AP4CH) views and 3 short-axis views at basal, midventricular and apical LV levels at end-diastole. Following the definition of the lateral and septal edges of the LV - mitral annulus and endocardial surface of the apical LV, a sequential analysis was conducted in order to create a 3D echocardiographic LV cast. Moreover, AP2CH and AP4CH views were helped to find the optimal TA level on the C7 short-axis view. Directly before tricuspid valve closure at end-diastole and directly before tricuspid valve opening at end-systole, the following TA morphological and functional parameters were calculated (Figure 2) [8,11,19].

### 2.1. Parameters Featuring TA Morphology

− TA diameter (TAD), evaluated by drawing a perpendicular line from the peak of TA curvature to the middle of the straight TA border;
− TA area (TAA), evaluated via planimetry;
− TA perimeter (TAP), evaluated via planimetry.

### 2.2. Parameters Featuring TA Function

− TA fractional shortening (TAFS), defined as ([end-diastolic TAD − end-systolic TAD]/end-diastolic TAD) × 100;
− TA fractional area change (TAFAC), defined as ([end-diastolic TAA − end-systolic TAA]/end-diastolic TAA) × 100.

### 2.3. Statistical Analysis

Continuous data were presented as average ± standard deviation (SD), while categorical data were demonstrated as *n* (%). Statistical significance was considered to be present when *p* < 0.05. Levene’s test was accomplished for assessing homogeneity of variances, while the Shapiro–Wilks test was used to test whether variables were normally distributed. Student’s *t*-test was used for normally distributed datasets, while the Mann–Whitney–Wilcoxon test was performed for non-normally distributed datasets. Pearson’s coefficients were measured to characterize correlations between variables. SPSS software (SPSS Inc., Chicago, IL, USA) was used for the statistical analyses.

## 3. Results

Demographic data. Two subgroups of healthy subjects were compared with each other: 29 subjects with TAPSE between 17 and 21 mm (average age: 27.2 ± 6.4 years, 16 men) were compared to 90 subjects with TAPSE ≥ 22 mm (average age: 30.0 ± 11.1 years, 33 men).

M-mode and two-dimensional Doppler echocardiography. In Table 1, the routine echocardiographic data of healthy subjects are presented. None of the subjects showed larger than grade 1 valvular insufficiency or significant valvular stenosis in any valve. No routine echocardiographic data differed between the subgroups.

Three-dimensional echocardiography. Three-dimensional echocardiography-derived LV volumes, mass and EF together with TA dimensions and functional properties are presented in Table 2 and Table 3. LV volumetric parameters did not differ between the subgroups. Subjects with TAPSE between 17 and 21 mm had tendentiously dilated TA dimensions as compared with subjects with TAPSE ≥ 22 mm. A significant difference was detected in the end-systolic TA area, leading to impaired TAFAC in subjects with lower TAPSE (17–21 mm).

Correlations. TAPSE did not show correlations with any TA sizes or ”sphincter-like” functional properties as assessed using 3DE.

Feasibility of TA measurements using 3DE and M-mode echocardiography. This study comprised 154 healthy adults, but due to insufficient image quality, 35 cases had to be excluded. Therefore, the overall feasibility was 77.3%.

Reproducibility data of 3DE-derived TA assessments. The mean ± SD of the differences in values measured by two examiners proved to be 0.03 ± 0.20 cm and 0.02 ± 0.39 cm for end-diastolic and end-systolic TAD, 0.03 ± 0.70 cm^2^ and −0.04 ± 0.69 cm^2^ for end-diastolic and end-systolic TAA and −0.10 ± 0.61 cm and 0.05 ± 0.60 cm for end-diastolic and end-systolic TAP with a correlation coefficient of 0.96, 0.96, 0.97, 0.96, 0.96 and 0.96 (*p* < 0.0001 for all), respectively (interobserver agreement). Similarly, the mean ± SD of the differences in values measured two times by the same observer was 0.02 ± 0.21 cm and −0.03 ± 0.17 cm for end-diastolic and end-systolic TAD, −0.02 ± 1.18 cm^2^ and −0.03 ± 0.38 cm^2^ for end-diastolic and end-systolic TAA and −0.04 ± 0.73 cm and 0.07 ± 0.58 cm for end-diastolic and end-systolic TAP with a correlation coefficient of 0.95, 0.96, 0.95, 0.96, 0.96 and 0.97 (*p* < 0.0001 for all), respectively (intraobserver agreement).

## 4. Discussion

In recent decades, there has been a huge development in cardiovascular imaging due to the technological advances characterizing this period. In addition to magnetic resonance imaging and computer tomography becoming accessible and important imaging methods in cardiology, new echocardiographic methods have emerged and become part of the daily routine. Two-dimensional speckle-tracking echocardiography (2D-STE) is a widely used method for the quantitative characterization of wall movements using strain parameters. Its advantage lies in its simplicity, and it is an option available in most modern devices. The prognostic value of LV global longitudinal strain calculated with 2D-STE has been confirmed [17]. Despite the above facts, it is theoretically not optimal, since it only measures in a given plane and therefore does not take into account regional differences [20]. Three-dimensional echocardiography is used to examine the heart and its cavities in 3D using virtually created 3D models that take into account the cardiac cycle. Three-dimensional speckle-tracking echocardiography (3D-STE) combines the advantages of these two methods. While it allows the heart to be seen in 3D, with the help of the digitally acquired 3D echocardiographic “echocloud”, it can measure not only the strain values, but also the rotational parameters at the same time as volumetric measurements. These advantages make 3D-STE the most modern echocardiographic method that is currently available, even though it has some limitations (e.g., problems with image quality) [4,5,6,7,8,9,10,11,12,17]. Despite the above advantages, 3D-STE is still not as widespread as 2D-STE or volumetric 3DE. Recent scientific findings have shown that 3DE is capable of measuring changes in annular size and calculating the functional parameters of the atrioventricular valves, taking into account the cardiac cycle. These functional parameters only characterize the sphincter-like function of the mitral or tricuspid valve calculated from changes in size (2D-projected diameter and area) of the given valve [8,9,10,11]. However, these valves are formations with a spatial complex structure, having spatial movement. Longitudinal spatial displacement is also present in the case of these valves, being characterized by a well-known and commonly used parameter called TAPSE [12].

Owing to the advantages of 3DE detailed above, this method is suitable for performing physiological studies by calculating several parameters at the same time using a digitally acquired 3DE database. In our present study, the LV volumes and the LV-EF were determined during 3DE along with the annular data of TV and the TAFAC and TAFS parameters characterizing the sphincter-like function of TA. Moreover, TAPSE was measured at the same time as the 3DE [4,5,6,7,8,9,10,11,20].

In a healthy person, the TA is a saddle-shaped dynamic structure, the expansion of which is accompanied by a change in its shape; as a result, it becomes more circular and planar [1]. In the event of backflow through the TV during systole, tricuspid regurgitation is present. Tricuspid regurgitation is organic in origin only in 10–15% of cases; most patients have FTR due to a distorted RV, papillary muscle/chordae or TA, with structurally normal leaflets [1,2,3]. FTR is mostly due to LV dysfunction, aortic or mitral valve disease or pulmonary vascular or interstitial disorders and is accompanied by consequent pulmonary hypertension [20,21]. FTR is considered to be “classical” or ventricular if it is secondary to TA enlargement and tethering of the leaflets and is associated with RV dilation/dysfunction. Previously, if there was no pulmonary hypertension or left heart disorder, FTR was considered to be idiopathic tricuspid regurgitation, which is closely associated with age and atrial fibrillation (AF). Recently, a new concept has been introduced, the so-called atrial FTR. This can be seen in the presence of AF, when RA enlargement and dysfunction lead to the dilation of the TA, leaflet malcoaptation and loss of TA sphincter-like function. Apparently, in these cases, RA dilation plays a greater role in TA dilation and FTR than the RV [3,19,21,22].

Evaluation of TAPSE is a well-known, old-fashioned, simple M-mode echocardiographic method with a significant prognostic value. It is easy to reproduce as a one-plane measurement of TA function in a longitudinal direction featuring its longitudinal displacement [12]. TAPSE is often used as an echocardiographic measurement of RV systolic function and a surrogate of the RV strain as well. TAPSE correlates with and predicts RV-EF [14]. TAPSE < 17 mm proved to have acceptable specificity in separating pathological conditions from healthy subjects [12,15], while its mean value was 21.7 ± 2.8 mm, according to a recent study, which is a lower value than previously described [12,13]. Abnormal TAPSE can be detected in pulmonary hypertension, RV ischemia, heart failure and congenital heart diseases [12,13,15].

Normal reference values for TA derived from 2D echocardiography and cardiac magnetic resonance imaging are available [23,24]. However, 3DE is the method of choice for non-invasive TA assessments as well [8,9,10,25,26,27,28,29]. Although this method is not widely used, mitral and tricuspid annuli could be easily assessed ”en-face” following plane optimizations on AP2CH and AP4CH views with planimetry with respect to the cardiac cycle [4,5,6,7,8,9,10,11]. TAFAC and TAFS are quantitative features of TA’s ”sphincter-like" function during the cardiac cycle [25,26].

Non-invasive cardiovascular imaging technologies, including echocardiography, are developing rapidly in the XXIst century, allowing more detailed non-invasive morphologic and functional analysis of not only of the atria and the ventricles, but the valves as well. Moreover, these imaging techniques have become part of the daily routine of cardiologists. Three-dimensional echocardiography is a good example of this enormous technological advancement, allowing detailed analysis of cardiac mechanics [4,5,6,7]. However, a better understanding of the methods revealed some previously unknown problems. One such problem is that LV volumes measured with 2D echocardiography and those determined with 3DE are not interchangeable [30,31]. Our own presented results highlight this problem as well, since the LV volumes measured with different methods are different; the LV-EDV values measured with 3DE are lower. As a consequence, LV-EF is lower as well [30,31]. However, 3DE is reliable in determining LV-EF [31]. Based on international recommendations, the cut-off value for LV-EF determined using 2D echocardiography is ≥55% [17], while based on literature data, the cut-off value is around 47–55%, as measured using 3DE and considering age and gender [31]. Our results, presented herein, are in accordance with these facts.

In the present study, 3DE-derived TA dimensions and TAFAC were demonstrated to be associated with TAPSE assessed via M-mode echocardiography in healthy subjects without FTR. Lower TAPSE was associated with lower TAFAC, which was related to more dilated end-systolic TAA. However, direct correlations between TA functional features could not be demonstrated. This result suggests a relationship between echocardiographic TA dimensions and TA features for “sphincter-like” (TAFAC) and “longitudinal” (TAPSE) functions. These results could suggest sensitive and harmonic cooperative ”sphincter-like” and “longitudinal” TA functions in healthy adults before FTR develops. However, other studies are required to confirm the presented results and to demonstrate abnormalities in the ”sphincter-like” (TAFAC) and “longitudinal” (TAPSE) properties of TA function in different disorders with FTR.

## 5. Limitations

The following limitations arose during assessments:

- Although the TA has a characteristic spatial saddle shape, only its 2D-projected image was analyzed [1,2].
- The image quality of echocardiographic analysis is an important issue, still being worse in the case of 3DE than in the case of 2D echocardiography, which should be taken into account when interpreting the findings. 3DE has several technical difficulties, including lower frame rate and larger transducer size, which can significantly affect image quality. Nevertheless, considering both the advantages and disadvantages, the clinical role of 3DE is unquestionable [5,6,7].
- Three-dimensional echocardiography-derived image quality is also highly dependent on stitching and motion artifacts [5,6,7].
- This study did not compare 2D echocardiography versus 3DE in the measurement of TA.
- Three-dimensional echocardiography-derived chamber quantifications of atria and ventricles were also not performed in this study.
- Validation of our 3DE results using other imaging methods could have further strengthened the significance of our scientific findings. Similar studies may be the subject of clinical trials in the future.
- STE-derived featuring of the TA function was not purposed either.
- As FTR was assessed visually, using a more advanced quantification technique would have strengthened our findings [1,2,3].
- Healthy subjects were involved in this study. However, neither special laboratory tests nor imaging testing were performed to completely exclude disorders in the early stages.

## 6. Conclusions

Three-dimensional echocardiography is capable of measuring the TA’s dimensions and functional ”sphincter-like” properties, which are associated with TAPSE, suggesting a sensitive and harmonic TA function in healthy adults without FTR.

## Figures and Tables

**Table 1:** Two-dimensional Doppler echocardiographic data of healthy subjects.

Parameters	Subjects (*n* = 119)	TAPSE 17–21 mm (*n* = 29)	TAPSE ≥22 mm (*n* = 90)
LA diameter (mm)	36.9 ± 3.3	37.2 ± 3.4	36.8 ± 3.2
LV end-diastolic diameter (mm)	48.1 ± 3.6	47.8 ± 3.8	48.3 ± 3.6
LV end-diastolic volume (mL)	105.8 ± 24.0	100.4 ± 28.1	107.7 ± 22.2
LV end-systolic diameter (mm)	32.4 ± 3.5	31.9 ± 3.2	32.6 ± 3.6
LV end-systolic volume (mL)	38.3 ±9.6	36.9 ± 9.0	38.9 ± 9.8
Interventricular septum (mm)	9.1 ± 1.2	8.9 ± 1.2	9.2 ± 1.2
LV posterior wall (mm)	9.3 ± 1.4	9.4 ± 1.6	9.3 ± 1.4
LV ejection fraction (%)	64.5 ±4.3	64.9 ± 3.3	64.3 ± 4.6

Abbreviations: LA = left atrial, LV = left ventricular, TAPSE = tricuspid annular plane systolic excursion.

**Table 2:** Comparison of three-dimensional-echocardiography-derived left ventricular parameters in healthy subjects.

Data	Subjects (*n* = 119)	TAPSE 17–21 mm (*n* = 29)	TAPSE ≥22 mm (*n* = 90)
LV-EDV (mL)	85.8 ± 20.8	82.4 ± 23.8	87.0 ± 19.7
LV-ESV (mL)	36.1 ± 10.2	34.4 ± 12.2	36.7 ± 9.4
LV-EF (%)	58.0 ± 5.7	58.8 ± 6.6	57.7 ± 5.4
LV mass (g)	164 ± 32	161 ± 28	165 ± 33

Abbreviations: LV = left ventricular, EDV = end-diastolic volume, ESV = end-systolic volume, EF = ejection fraction.

**Table 3:** Comparison of three-dimensional-echocardiography-derived tricuspid annular morphological and functional parameters in healthy subjects.

Data	Subjects (*n* = 119)	TAPSE 17–21 mm (*n* = 29)	TAPSE ≥22 mm (*n* = 90)
Tricuspid annular dimensions			
TAD-D (cm)	2.55 ± 1.91	2.43 ± 0.40	2.10 ± 2.19
TAA-D (cm^2^)	7.53 ± 1.66	7.70 ± 1.91	5.70 ± 1.58
TAP-D (cm)	10.57 ± 1.18	10.63 ± 1.22	9.30 ± 1.17
TAD-S (cm)	1.84 ± 0.29	1.90 ± 0.40	1.70 ± 0.25
TAA-S (cm^2^)	5.41 ± 1.43	5.85 ± 1.90	3.70 ± 1.22 *
TAP-S (cm)	9.11 ± 1.11	9.32 ± 1.30	7.70 ± 1.05
Tricuspid annular ”sphincter-like” functional parameters			
TAFAC (%)	28.1 ± 9.2	24.8 ± 9.0	35.1 ± 9.1 *
TAFS (%)	23.0 ± 10.9	21.8 ± 10.2	19.1 ± 11.1
Tricuspid annular longitudinal functional parameter			
TAPSE (mm)	23.8 ± 2.9	20.2 ± 0.9	23.0 ± 2.3 *

Abbreviations: TAA-D = end-diastolic tricuspid annular area, TAA-S = end-systolic tricuspid annular area, TAD-D = end-diastolic tricuspid annular diameter, TAD-S = end-systolic tricuspid annular diameter, TAFAC = tricuspid annular fractional area change, TAFS = tricuspid annular fractional shortening, TAP-D = end-diastolic tricuspid annular perimeter, TAP-S = end-systolic tricuspid annular perimeter, TAPSE = tricuspid annular plane systolic excursion. * *p* < 0.05 vs. TAPSE 17–21 mm.

## Data Availability

The manuscript has not been published elsewhere.

## References

[1] Putthapiban P., Amini M.R., Abudayyeh I. (2022). Anatomy of the Tricuspid Valve and Pathophysiology of Tricuspid Regurgitation. Interv. Cardiol. Clin..

[2] Tadic M., Cuspidi C., Morris D.A., Rottbauer W. (2022). Functional tricuspid regurgitation, related right heart remodeling, and available treatment options: Good news for patients with heart failure?. Heart Fail. Rev..

[3] Badano L.P., Muraru D., Enriquez-Sarano M. (2013). Assessment of functional tricuspid regurgitation. Eur. Heart J..

[4] Franke A., Kuhl H.P. (2003). Second-generation real-time 3D echocardiography: A revolutionary new technology. MedicaMundi.

[5] Nemes A., Kalapos A., Domsik P., Forster T. (2012). Three-dimensional speckle-tracking echocardiography—A further step in non-invasive three-dimensional cardiac imaging. Orv. Hetil..

[6] Ammar K.A., Paterick T.E., Khanderia B.K., Jan M.F., Kramer C., Umland M.M., Tercius A.J., Baratta L., Tajik A.J. (2012). Myocardial mechanics: Understanding and applying three-dimensional speckle tracking echocardiography in clinical practice. Echocardiography.

[7] Urbano-Moral J.A., Patel A.R., Maron M.S., Arias-Godinez J.A., Pandian N.G. (2012). Three-dimensional speckle-tracking echocardiography: Methodological aspects and clinical potential. Echocardiography.

[8] Nemes A., Kormányos Á., Rácz G., Ruzsa Z., Ambrus N., Lengyel C. (2023). Normal reference values of tricuspid annular dimensions and functional properties in healthy adults using three-dimensional speckle-tracking echocardiography (insights from the MAGYAR-Healthy Study). Quant. Imaging Med. Surg..

[9] Volpato V., Mor-Avi V., Veronesi F., Addetia K., Yamat M., Weinert L., Genovese D., Tamborini G., Pepi M., Lang R.M. (2020). Three-dimensional echocardiography investigation of the mechanisms of tricuspid annular dilatation. Int. J. Cardiovasc. Imaging..

[10] Bieliauskienė G., Kažukauskienė I., Kramena R., Zorinas A., Mainelis A., Zakarkaitė D. (2023). Three-dimensional analysis of the tricuspid annular geometry in healthy subjects and in patients with different grades of functional tricuspid regurgitation. Cardiovasc. Ultrasound..

[11] Nemes A., Rácz G., Kormányos Á. (2022). Tricuspid Annular Abnormalities in Isolated Left Ventricular Non-compaction-Insights From the Three-dimensional Speckle-Tracking Echocardiographic MAGYAR-Path Study. Front. Cardiovasc. Med..

[12] Rudski L.G., Lai W.W., Afilalo J., Hua L., Handschumacher M.D., Chandrasekaran K., Solomon S.D., Louie E.K., Schiller N.B. (2010). Guidelines for the echocardiographic assessment of the right heart in adults: A report from the American Society of Echocardiography endorsed by the European Association of Echocardiography, a registered branch of the European Society of Cardiology, and the Canadian Society of Echocardiography. J. Am. Soc. Echocardiogr..

[13] Nel S., Nihoyannopoulos P., Libhaber E., Essop M.R., Dos Santos C.F., Matioda H., Waterworth C., Grinter S., Meel R., Peters F. (2020). Echocardiographic Indices of the Left and Right Heart in a Normal Black African Population. J. Am. Soc. Echocardiogr..

[14] Sato T., Tsujino I., Ohira H., Oyama-Manabe N., Yamada A., Ito Y.M., Goto C., Watanabe T., Sakaue S., Nishimura M. (2012). Validation study on the accuracy of echocardiographic measurements of right ventricular systolic function in pulmonary hypertension. J. Am. Soc. Echocardiogr..

[15] Tamborini G., Pepi M., Galli C.A., Maltagliati A., Celeste F., Muratori M., Rezvanieh S., Veglia F. (2007). Feasibility and accuracy of a routine echocardiographic assessment of right ventricular function. Int. J. Cardiol..

[16] Ghio S., Recusani F., Klersy C., Sebastiani R., Laudisa M.L., Campana C., Gavazzi A., Tavazzi L. (2000). Prognostic usefulness of the tricuspid annular plane systolic excursion in patients with congestive heart failure secondary to idiopathic or ischemic dilated cardiomyopathy. Am. J. Cardiol..

[17] Lang R.M., Badano L.P., Mor-Avi V., Afilalo J., Armstrong A., Ernande L., Flasckampf F.A., Foster E., Goldstein S.A., Kuznetsova T. (2015). Recommendations for cardiac chamber quantification by echocardiography in adults: An update from the American Society of Echocardiography and the European Association of Cardiovascular Imaging. J. Am. Soc. Echocardiogr..

[18] Lancellotti P., Tribouilloy C., Hagendorff A., Popescu B.A., Edvardsen T., Pierard L.A., Badano L., Zamorano J.L. (2013). Scientific Document Committee of the European Association of Cardiovascular Imaging. Recommendations for the echocardiographic assessment of native valvular regurgitation: An executive summary from the European Association of Cardiovascular Imaging. Eur. Heart J. Cardiovasc. Imaging.

[19] Nemes A., Kormányos Á., Ruzsa Z., Achim A., Ambrus N., Lengyel C. (2023). Three-Dimensional Speckle-Tracking Echocardiography-Derived Tricuspid Annular Dimensions and Right Atrial Strains in Healthy Adults-Is There a Relationship? (Insights from the MAGYAR-Healthy Study). J. Clin. Med..

[20] Gual-Capllonch F., Cediel G., Ferrer E., Teis A., Juncà G., Vallejo N., López-Ayerbe J., Bayes-Genis A. (2021). Sex-Related Differences in the Mechanism of Functional Tricuspid Regurgitation. Heart Lung Circ..

[21] Florescu D.R., Muraru D., Volpato V., Gavazzoni M., Caravita S., Tomaselli M., Ciampi P., Florescu C., Bălșeanu T.A., Parati G. (2022). Atrial Functional Tricuspid Regurgitation as a Distinct Pathophysiological and Clinical Entity: No Idiopathic Tricuspid Regurgitation Anymore. J. Clin. Med..

[22] Muraru D., Addetia K., Guta A.C., Ochoa-Jimenez R.C., Genovese D., Veronesi F., Basso C., Iliceto S., Badano L.P., Lang R.M. (2021). Right atrial volume is a major determinant of tricuspid annulus area in functional tricuspid regurgitation: A three-dimensional echocardiography study. Eur. Heart J. Cardiovasc. Imaging.

[23] Dwivedi G., Mahadevan G., Jimenez D., Frenneaux M., Steeds R.P. (2014). Reference values for mitral and tricuspid annular dimensions using two-dimensional echocardiography. Echo. Res. Pract..

[24] Zhan Y., Debs D., Khan M.A., Nguyen D.T., Graviss E.A., Shah D.J. (2019). Normal Reference Values and Reproducibility of Tricuspid Annulus Dimensions Using Cardiovascular Magnetic Resonance. Am. J. Cardiol..

[25] Anwar A.M., Soliman O.I., Nemes A., van Geuns R.J.M., Geleijnse M.L., ten Cate F.J. (2007). Value of assessment of tricuspid annulus: Real-time three-dimensional echocardiography and magnetic resonance imaging. Int. J. Cardiovasc. Imaging.

[26] Anwar A.M., Geleijnse M.L., Soliman O.I., McGhie J.S., Frowijn R., Nemes A., van den Bosch A.E., Galema T.W., ten Cate F.J. (2007). Assessment of normal tricuspid valve anatomy in adults by real-time three-dimensional echocardiography. Int. J. Cardiovasc. Imaging.

[27] Muraru D., Gavazzoni M., Heilbron F., Mihalcea D.J., Guta A.C., Radu N., Muscogiuri G., Tomaselli M., Sironi S., Parati G. (2022). Reference ranges of tricuspid annulus geometry in healthy adults using a dedicated three-dimensional echocardiography software package. Front. Cardiovasc. Med..

[28] Addetia K., Muraru D., Veronesi F., Jenei C., Cavalli G., Besser S.A., Mor-Avi V., Lang R.M., Badano L.P. (2019). 3-Dimensional Echocardiographic Analysis of the Tricuspid Annulus Provides New Insights Into Tricuspid Valve Geometry and Dynamics. JACC Cardiovasc. Imaging.

[29] Muraru D., Hahn R.T., Soliman O.I., Faletra F.F., Basso C., Badano L.P. (2019). 3-Dimensional Echocardiography in Imaging the Tricuspid Valve. JACC Cardiovasc. Imaging.

[30] Kleijn S.A., Aly M.F.A., Terwee C.B., van Rossum A.C., Kamp O. (2012). Reliability of left ventricular volumes and function measurements using three-dimensional speckle tracking echocardiography. Eur. Heart J. Cardiovasc. Imaging.

[31] Wood P.W., Choy J.B., Nanda N.C., Becher H. (2014). Left ventricular ejection fraction and volumes: It depends on the imaging method. Echocardiography.

